# Whole tumor RNA-sequencing and deconvolution reveal a clinically-prognostic PTEN/PI3K-regulated glioma transcriptional signature

**DOI:** 10.18632/oncotarget.17193

**Published:** 2017-04-18

**Authors:** Yuan Pan, Erin C. Bush, Joseph A. Toonen, Yu Ma, Anne C. Solga, Peter A. Sims, David H. Gutmann

**Affiliations:** ^1^ Department of Neurology, Washington University School of Medicine, St. Louis, MO, USA; ^2^ Departments of Systems Biology and of Biochemistry and Molecular Biophysics, Columbia University Medical Center, New York, NY, USA

**Keywords:** astrocytoma, neurofibromatosis, AKT, glioma stem cell, microglia

## Abstract

The concept that solid tumors are maintained by a productive interplay between neoplastic and non-neoplastic elements has gained traction with the demonstration that stromal fibroblasts and immune system cells dictate cancer development and progression. While less studied, brain tumor (glioma) biology is likewise influenced by non-neoplastic immune system cells (macrophages and microglia) which interact with neoplastic glioma cells to create a unique physiological state (glioma ecosystem) distinct from that found in the normal tissue. To explore this neoplastic ground state, we leveraged several preclinical mouse models of neurofibromatosis type 1 (NF1) optic glioma, a low-grade astrocytoma whose formation and maintenance requires productive interactions between non-neoplastic and neoplastic cells, and employed whole tumor RNA-sequencing and mathematical deconvolution strategies to characterize this low-grade glioma ecosystem as an aggregate of cellular and acellular elements. Using this approach, we demonstrate that optic gliomas generated by altering the germline *Nf1* gene mutation, the glioma cell of origin, or the presence of co-existing genetic alterations represent molecularly-distinct tumors. However, these optic glioma tumors share a 25-gene core signature, not found in normal optic nerve, that is normalized by microglia inhibition (minocycline), but not conventional (carboplatin) or molecularly-targeted (rapamycin) chemotherapy. Lastly, we identify a genetic signature conferred by Pten reduction and corrected by PI3K inhibition. This signature predicts progression-free survival in patients with either low-grade or high-grade glioma. Collectively, these findings support the concept that gliomas are composite ecological systems whose biology and response to therapy may be best defined by examining the tumor as a whole.

## INTRODUCTION

Histological examination of most solid tumors reveals that these cancers are composed of numerous distinct cell types, which are embedded in a rich extracellular matrix containing growth factors and cytokines. Among the cell types in these tumors are differentiated and stem-like neoplastic cells, as well as non-neoplastic cells, such as monocytes, fibroblasts, and endothelial cells [1]. With the recognition that the biological behaviors of many solid cancers are dictated by the productive interactions between these diverse cellular and acellular elements, the concept of a cancer ecosystem has been advanced [2–4]. In support of this concept, non-neoplastic stromal cells, such as fibroblasts and immune system cells, have been shown to drive solid tumor development and progression [5, 6]. Within the central nervous system, brain tumor (glioma) growth in experimental mouse models is similarly controlled by specific monocyte populations, including microglia and macrophages, that are recruited by the tumor cells through the elaboration of chemokines, like colony stimulating factor 1 (CSF-1) [7]. Additionally, productive interactions between monocytes and glioma cells create a feed-forward paracrine circuit that perpetuates high-grade glioma growth [8–10]. These studies in high-grade malignancies have suggested alternative approaches to cancer treatment, in which targeted therapies against stromal cell types or signals might emerge as a potential adjuvant approaches [11–15].

One of the clinical situations where stromal therapies might be particularly useful is in the treatment of pediatric low-grade tumors. Unlike adult high-grade malignancies, these tumors are slow growing (with proliferative indices often less than 1%) and arise in children whose brains are still developing. Therapies that target unique elements of the tumor ecosystem, not shared by the developing brain, would be highly desirable. This issue is well illustrated by the Neurofibromatosis type 1 (NF1) cancer predisposition syndrome, in which affected children are prone to the formation of low-grade nervous system tumors (peripheral nerve sheath tumors and optic pathway gliomas). Examination of these tumors reveals prominent immune system infiltration and interdependencies between neoplastic and non-neoplastic cell types [16–22]. Specifically, the formation and continued growth (maintenance) of low-grade optic gliomas in mice require a supportive tumor microenvironment containing microglia, which are attracted to the developing cancer by chemokines [20], and elaborate specific growth factors that promote neoplastic cell growth [19]. These cellular interactions could be hypothesized to create a new homeostatic state, not present in the non-neoplastic optic nerve, and thus establish new circuitries and interdependencies for chemotherapeutic targeting.

In the current study, we employed whole-tumor RNA-sequencing and mathematical deconvolution strategies using preclinical mouse models of *Nf1* optic glioma to characterize the tumor ecosystem as an aggregate gene expression pattern distinct from the healthy optic nerve. Leveraging numerous different murine optic glioma genetically-engineered mouse (GEM) models, we found that histologically-similar tumors are molecularly distinct. However, despite their uniqueness, all *Nf1* murine optic gliomas examined share a 25-gene expression signature that distinguishes tumor tissue from normal optic nerve. This core genetic signature was minimally affected by conventional (carboplatin) or biologically-targeted (rapamycin) therapy, but was normalized following minocycline treatment, which inhibits tumor-associated microglia function. Lastly, we discovered a PTEN/PI3K-driven molecular signature that correlates with the overall survival of patients with either low-grade or high-grade glioma. Taken together, our study suggests that shared and unique whole-tumor gene expression patterns may serve as biomarkers of tumor response to therapy and patient survival.

## RESULTS AND DISCUSSION

### Whole tumor RNA-sequencing of a diverse collection of *Nf1* optic glioma tumors reveals molecularly-distinct neoplasms

**Figure 1:**
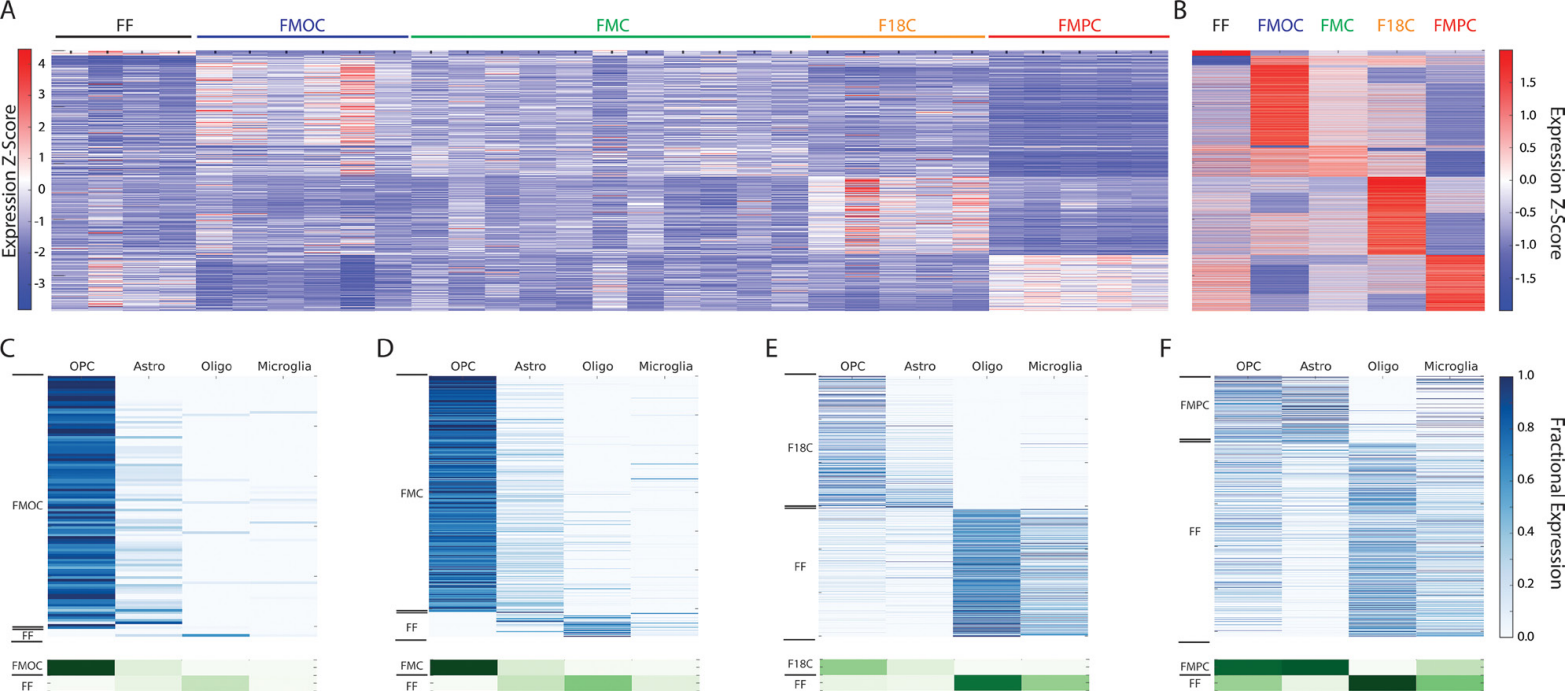
(**A**) Heatmap of RNA-Seq expression z-scores computed for all genes that are differentially expressed (p_adj_ < 0.01, |log_2_(fold-change)| > 1) between all pairwise comparisons of the optic glioma models and the healthy optic nerve (FF) controls. Each column in the heatmap is an individual sample. (**B**) Same as (A), but averaged over all samples for each genotype. (**C**) Computational deconvolution of cell type-specific gene expression for genes that are differentially expressed between FMOC tumors and FF controls. In the top panel, each row in the heatmap corresponds to a specific differentially-expressed gene and each column corresponds to a cell type. The lower panel shows the aggregate expression in each cell type for all genes that are high in FMOC or FF tumors. The analysis shows that the FMOC tumors are highly enriched in OPC-like gene expression. (**D**) Same as (C), but for differentially-expressed genes from the comparison of FMC tumors to FF controls. Similar to FMOC, the FMC tumors are also dominated by an OPC-like gene expression pattern. (**E**) Same as (C), but for differentially-expressed genes from the comparison of F18C tumors to FF controls. The F18C tumors have both higher OPC-like and astrocytic gene expression relative to FF controls. (**F**) Same as (C), but for differentially-expressed genes from the comparison of FMPC tumors to FF controls, demonstrating a more pronounced astrocytic gene expression in FMPC gliomas. OPC, oligodendrocyte progenitor cell; Astro, astocyte; Oligo, oligodendrocyte.

In an effort to accurately represent the heterogeneity inherent in human NF1-associated optic pathway glioma (NF1-OPG), we have previously engineered mice that differ with respect to the germline *Nf1* gene mutation (null allele (FMC) [23] versus NF1 patient germline *NF1* gene mutation; R681* (F18C) [24]), the cell of origin (GFAP^+^ neuroglial progenitors (FMC) [23] versus Olig2^+^ progenitor cells (FMOC) (Solga A, manuscript in preparation)), and the presence of a co-existing heterozygous *Pten* deletion in the tumor cells (FMPC) [25, 26]. Using these four optic glioma strains, we performed whole tumor RNA-Seq on 27 specimens along with four normal age- and sex-matched optic nerves. Gene expression heatmap representation of all 31 samples across 4,624 genes revealed differentially-expressed gene sets in at least one pairwise comparison relative to normal optic nerve, as well as between *Nf1* GEM optic glioma models (Figure 1A). While some sample-to-sample heterogeneity was found between whole tumor specimens from the same GEM model, significantly larger differences among the four models were demonstrated by the average profiles (Figure 1B). Because these expression profiles were from homogenized whole tumor tissue, the observed molecular differences between models likely reflect contributions from multiple distinct cell types. However, despite clear differences in tumor latency, proliferative rates, and tumor volumes, immunohistochemical analyses of these tumors revealed only slight differences in the percentages of microglia (Iba1^+^ cells), astroglial (GFAP^+^ or S100β^+^ cells), or Olig2^+^ progenitor cells [23–25].

To better understand the murine *Nf1* optic glioma ecosystem as a whole, we next sought to estimate the cell type-specific contributions to the observed gene expression differences. In previous studies, we and others have used computational approaches to deconvolve the data into potential cell type profiles. Whole transcriptome deconvolution requires two basic inputs - genome-wide transcript abundances and a measure of cellular composition - both of which can be derived from RNA-Seq data. As demonstrated in our previous study, in which we applied deconvolution to human high-grade gliomas, we can use the abundance of a cell type-specific marker or set of markers for each cell type in any given tissue to estimate the fractional composition of each cell type [27, 28]. Hence, we are able to use the RNA expression levels of cell type-specific marker genes in the composite profiles of each specimen to solve for an estimated abundance of each gene in each cell type by least-squares regression. While this approach assumes that cell type-specific RNA abundances are invariant across specimens, the fitting procedure allows us to restrict our analysis to genes with expression patterns that are fit well by this model.

For these analyses, we specifically focused on glial cell populations (microglia and macroglia [oligodendrocytes, astrocytes, oligodendrocyte precursors]; Figure 1C–1F). As such, the glial cell type-specific contributions for genes that are differentially expressed (*p*_adj_<0.01, |log_2_(fold-change)| > 1) between each *Nf1* GEM optic glioma model and normal optic nerve can be represented, as estimated by computational deconvolution. Using this approach, several important observations were made: First, all of the *Nf1* GEM optic glioma models showed significant depletion of genes specific to mature oligodendrocytes compared to normal optic nerve (FF). Second, while all four models exhibited enrichment of oligodendrocyte progenitor cell (OPC) gene expression, this finding was most pronounced in the FMOC and FMC optic glioma models relative to the F18C and FMPC optic glioma models. Third, the largest distinction between the FMPC optic glioma model and the normal optic nerve was enrichment of astrocyte gene expression. This result suggests that *Pten* reduction, which is the only genetically-engineered difference between the FMC and FMPC optic glioma models, may alter the glial lineage character of the tumor. However, these changes in gene expression did not correlate with changes in Olig2^+^, NG2^+^ or GFAP^+^ cell numbers between *Nf1* GEM optic glioma strains ([24, 25]; Supplementary Figure 1). The apparent disconnect between glial cell-estimated gene expression and actual glial cell numbers suggests that these differentially-expressed gene signatures reflect global changes in the tumor ecosystem, rather than alterations in overall tumor cellular composition. Moreover, analysis of previously-acquired RNA-sequencing data from FMC tumor-associated microglia relative to those from *Nf1*+/− mice [19] demonstrated that the differentially-expressed genes assigned to microglia by deconvolution were not similarly altered in the actual microglia from these tumors (data not shown). Collectively, these results support the notion that the gene expression patterns in the tumor do not reflect the individual contributions of each cell type, but rather the composite interactions of all cell types.

### All *Nf1* optic glioma GEM models share a core gene expression signature that is minocycline-sensitive

**Figure 2:**
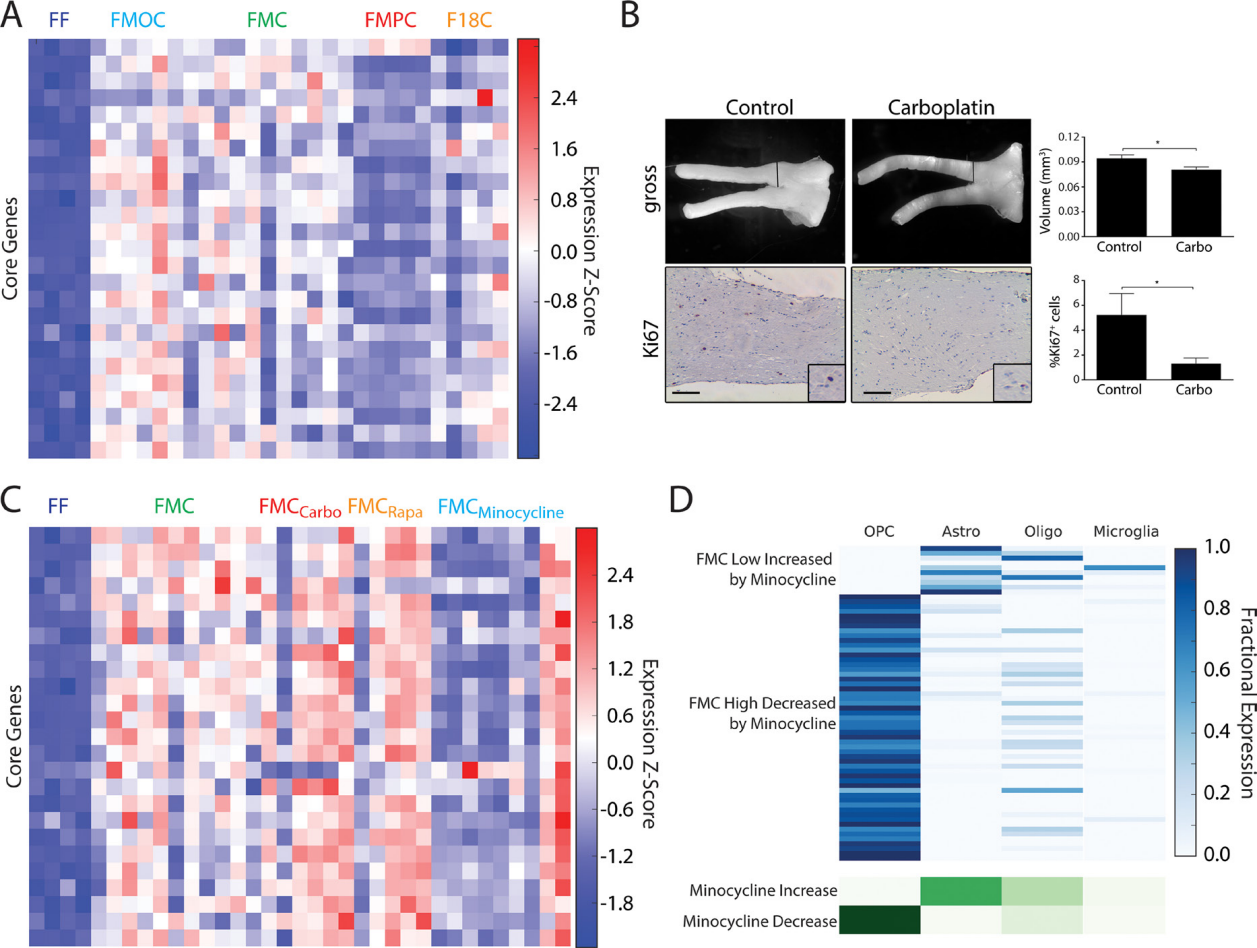
(**A**) Heatmap of RNA-Seq expression z-scores computed for the 25-gene core signature that is commonly differentially upregulated across all optic glioma models. (**B**) FMC mice treated with carboplatin at 3 months of age, and analyzed 1 month later, reveals decreased tumor volumes and percentages of Ki67^+^ cells. Control mice were treated with vehicle only and processed identically. *p < 0.05. Scale bar, 100 μm. (**C**) Same as (A), but for minocycline-treated and untreated FMC gliomas and FF controls. (**D**) Deconvolution of genes that are normalized in FMC by minocycline (highly or lowly expressed in FMC gliomas vs. FF controls, but either decreased or increased, respectively, by minocycline treatment).

While Figure 1 highlights the molecular differences among the four different *Nf1* optic glioma models, all of these tumors shared a core gene expression signature. In this regard, we identified a 25-gene signature that is universally differentially expressed (increased) relative to normal optic nerves across the 27 optic glioma specimens (Table 1; Figure 2A). Interestingly, this signature was most pronounced in the FMOC and FMC tumors and relatively diminished in the FMPC tumors. Accordingly, our deconvolution model suggests that these genes are predominantly associated with OPC-like cells. While there are more GFAP^+^ cells in all *Nf1* optic glioma models relative to the non-neoplastic optic nerve, there are only small differences in the relative proportions of Olig2^+^, GFAP^+^, or NG2^+^ glial cells between the various GEM models ([24, 25]; Supplementary Figure 1). In addition, GO-term analysis of the core genes did not reveal enrichment in specific categories (data not shown). As before, we favor the interpretation that this core signature represents a “neoplastic state” gene expression network established by the glioma ecosystem. Since this core gene expression signature distinguishes normal from tumor-bearing optic nerve, we hypothesize that it might be used to identify brain tumor treatments that best normalize the glioma ecosystem.

**Table 1:** Genes comprising the 25-core signature in all optic glioma models

Gene	Name
*Aldh1l1*	aldehyde dehyrogenase 1 family member L1
*Btbd3*	BTB (POZ) domain-containing 3
*Btbd17*	BTB (POZ) domain-containing 17
*Cd38*	CD38
*Cspg5*	chondroitin sulfate proteoglycan 5 (Caleb)
*Cxcl14*	C-X-C motif chemokine ligand 14
*Elmo2*	engulfment and cell motility 2
*Etv5*	ets variant 5 (Erm)
*Fabp5*	fatty acid binding protein 5 (E-Fabp)
*Fgfrl1*	fibroblast growth factor receptor-like 1
*Gja1*	gap junction protein, alpha 1 (connexin-43)
*Igdcc4*	immunoglobulin superfamily DCC subclass member 4 (Nope)
*Marcks*	myristoylated alanine-rich protein kinase C substrate
*Ncan*	neurocan (Cspg3)
*Ntsr2*	neurotensin receptor 2
*Pcdhgc3*	protocadherin gamma subfamily C, 3
*Pygm*	glycogen phosphorylase, muscle associated
*S1pr1*	sphingosine-1-phosphate receptor 1 (Edg1)
*Sdc3*	syndecan 3
*Sfxn5*	sideroflexin 5
*Shc3*	SHC adaptor protein 3 (N-Shc)
*Slc6a11*	solute carrier family 6 member 11 (Gat3)
*Spry4*	sprouty homolog 4
*Tril*	TLR4 interactor with leucine-rich repeats
*Wnt7b*	wingless-type MMPTV integration site family, member 7B

To explore this idea further, we leveraged prior preclinical studies from our laboratory in which conventional and biologically-targeted chemotherapy agents were evaluated prior to clinical translation. While our previous preclinical experiments demonstrated that temozolomide (TMZ) is effective at inducing tumor death [29], we now employed carboplatin as a standard chemotherapy reference, as it is the first line therapy for children with NF1-OPG [30, 31]. Similar to that observed in FMC mice following TMZ treatment, carboplatin induced a significant reduction in both tumor volume and proliferation (%Ki67^+^ cells; Figure 2B). Additionally, we evaluated the effect of two biologically-targeted therapies previously shown to inhibit mouse *Nf1* optic glioma growth *in vivo*. We selected rapamycin (mechanistic target of rapamycin [mTOR] inhibitor; [29]) and minocycline (microglia inhibitor; [17]) to inhibit neoplastic and stromal cell function, respectively, in separate experiments.

Overall, we found that rapamycin treatment resulted in very few changes in the 25-core gene expression profile compared to carboplatin and minocycline. This result is not surprising in light of recent clinical studies on a related NF1-associated benign tumor, the plexiform neurofibroma, in which rapamycin analogs exhibited little clinical efficacy [32, 33]. Importantly, while carboplatin treatment clearly inhibited tumor growth *in vivo*, there was also little effect on the 25-core gene expression signature. This finding suggests that this time-tested chemotherapy may not normalize the tumor ecosystem. In this regard, tumor shrinkage is observed in the minority of children with NF1-OPG [34, 35], and, when observed, is often relatively modest or not longstanding (durable response) [36–38].

While neither anti-neoplastic cell therapy normalized the optic glioma core signature, minocycline treatment resulted in downregulation of this 25-core gene signature in the majority of mice (Figure 2C). Furthermore, analysis of the entire set of genes that were normalized by minocycline treatment using our deconvolution model showed that these alterations were largely associated with OPC-like cells, rather than microglia (Figure 2D). These results imply that blocking microglia function with minocycline has its greatest impact on the tumors themselves. This finding is consistent with recent RNA-sequencing studies on *Nf1* optic glioma-associated microglia, in which one chemokine (CCL5) made by these microglia positively regulates both microglia recruitment and glioma growth [19]. Future studies aimed at normalizing the tumor microenvironment using stroma-targeted treatments may prove most effective at re-establishing a normal tissue ecosystem, and result in more durable glioma responses in human clinical trials.

### A PTEN/PI3K-dependent gene signature is associated with poor glioma prognosis

**Figure 3:**
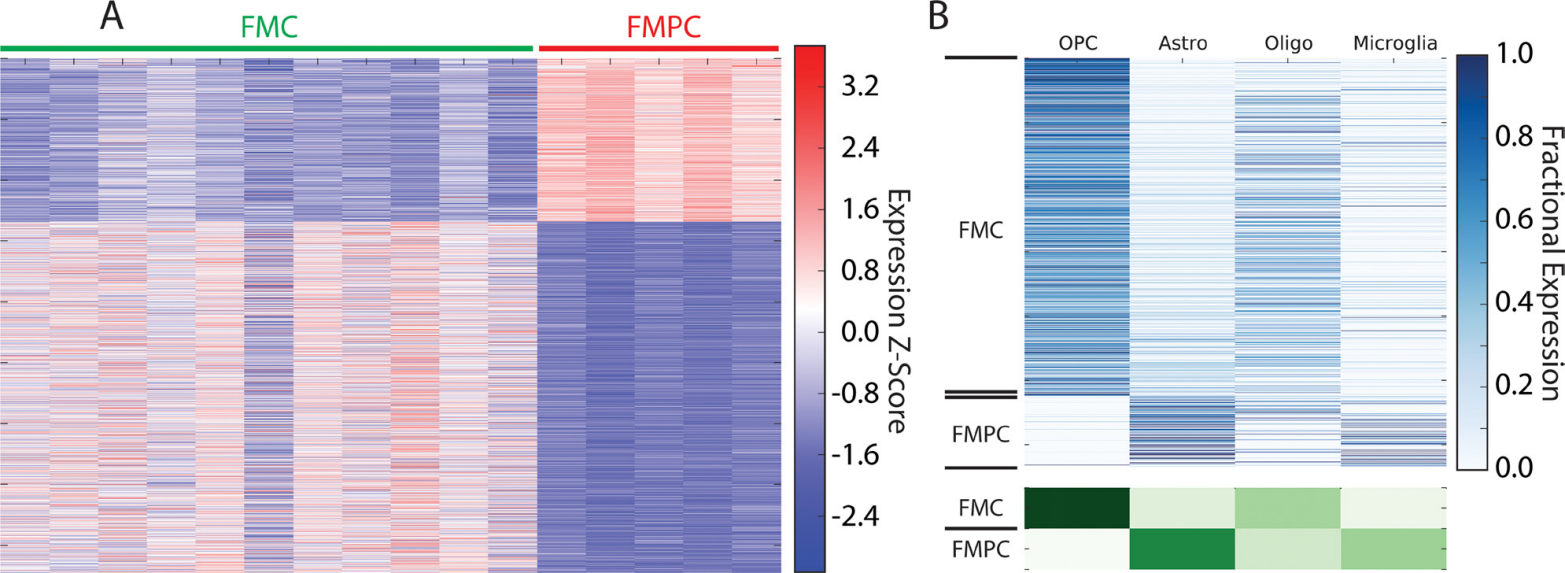
(**A**) Heatmap of RNA-Seq expression z-scores computed for genes that are differentially expressed (*p*_adj_ < 0.01, |log_2_(fold-change)| > 1) between FMPC and FMC gliomas. (**B**) Deconvolution of genes that are differentially expressed between FMPC and FMC gliomas. In the top panel, each row in the heatmap corresponds to a specific differentially expressed gene and in each column corresponds to a cell type. The lower panel shows the aggregate expression in each cell type for all genes that are high in FMPC or FMC. The analysis shows that the FMC tumors are highly enriched in OPC-like gene expression compared to FMPC tumors, which are highly enriched in astrocytic gene expression.

As noted above, *Pten* deletion altered both the lineage resemblance and core signature expression of the FMC optic glioma model. Heat map representation (Figure 3A) and deconvolution profiles (Figure 3B) revealed a differentially-expressed gene signature between FMC and FMPC specimens (Table 2). In the latter analysis, genes associated with the FMC and FMPC models were enriched in OPC-like and astrocytic gene expression, respectively. Importantly, these differentially-expressed signatures were not reflected in the relative abundance of either OPCs or astrocytes (Supplementary Figure 1).

**Table 2:** Genes differentially-expressed between FMC and FMPC optic gliomas

Genes with high expression in FMPC		Name
*A4galt*		alpha 1,4-galactosyltransferase
*Actb*		actin, beta
*Akap12*		A kinase anchor protein 12
*Alyref*		Aly/REF export factor
*Angptl2*		angiopoietin-like 2
*Anxa11*		annexin A11
*Cd248*		CD248 antigen, endosialin
*Chrd*		chordin
*Col3a1*		collagen, type III, alpha 1
*Ctgf*		connective tissue growth factor
*Cxcl16*		chemokine C-X-C motif ligand 16
*D630003M21Rik*		unknown
*Dbn1*		drebrin 1
*Dnaja4*		DnaJ heat shock protein family member A4
*Dpysl3*		dihydropyrimidinase-like 3
*Dusp1*		dual specificity phosphatase 1
*Ehd2*		EH-domain containing 2
*Gpr81*		hydrocarboxylic acid receptor 1
*Heyl*		hairy/enhancer-of-split related with YRPW motif-like
*Hspb1*		heat shock protein 1
*Htra3*		HtrA serine peptidase 3
*Kdelr3*		Lys-Asp-Glu-Leu endoplasmic reticulum protein retention receptor 3
*Krt19*		keratin 19
*Lama4*		laminin, alpha 4
*Lgals3*		galectin 3
*Lmna*		lamin A
*Loxl1*		lysyl oxidase-like 1
*Ltbp4*		latent transforming growth factor beta binding protein 4
*Mmp23*		matrix metallopeptidase 23
*Msc*		musculin
*Mvp*		major vault protein
*Mxra7*		matrix-remodeling associated 7
*Nbl1*		neuroblastoma, suppression of tumorigenicity 1
*Ntn1*		netrin 1
*Pam*		peptidylglycine alpha-amidating monooxygenase
*Pamr1*		peptidase domain containing associated with muscle regeneration 1
*Ptrf*		polymerase I and transcript release factor
*Rab3d*		RAB3D, member RAS oncogene family
*Rasgef1b*		RasGEF domain family, member 1B
*Rcn3*		reticulocalbin 3, EF-hand calcium binding domain
*Rnase1*		ribonuclease, RNase A family, 1
*Sema3b*		sema domain, immunoglobulin domain, short basic domain, secreted, 3B
*Serpinh1*		serine (or cysteine) peptidase inhibitor, clade H, member 1
*Spon2*		spondin 2
*Stk32c*		serine/threonine kinase 32C
*Tgfbi*		transforming growth factor, beta induced
*Tgm2*		transglutaminase 2, C polypeptide
*Tmem204*		transmembrane protein 204
*Tubb6*		tubulin, beta 6 class V
*1500009L16Rik*		unknown
**Genes with low expression in FMPC**	**Name**	
*Abat*	4-aminobutyrate aminotransferase	
*Abcd2*	ATP-binding cassette, sub-family D (ALD), member 2	
*Acsbg1*	acyl-CoA synthetase bubblegum family member 1	
*Agl*	amylo-1,6-glucosidase, 4-alpha-glucanotransferase	
*Apba2*	amyloid beta precursor protein-binding, family A, member 2	
*Aqp4*	aquaporin 4	
*Btbd17*	BTB (POZ) domain containing 17	
*Cadm1*	cell adhesion molecule 1	
*Chpt1*	choline phosphotransferase 1	
*Cmip*	c-Maf inducing protein	
*Cntn1*	contactin 1	
*Dmp1*	dentin matrix protein 1	
*Elavl3*	embryonic lethal, abnormal vision-like 3	
*Elovl2*	elongation of very long chain fatty acids-like 2	
*Elovl6*	ELOVL family member 6, elongation of long chain fatty acids	
*Frrs1l*	ferric-chelate reductase 1 like	
*Fut9*	fucosyltransferase 9	
*Gabrb1*	gamma-aminobutyric acid A receptor, subunit beta 1	
*Gnaq*	guanine nucleotide binding protein, alpha q polypeptide	
*Gria2*	glutamate receptor, ionotropic, AMPA2	
*Gria4*	glutamate receptor, ionotropic, AMPA4	
*Grin3a*	glutamate receptor ionotropic, NMDA3A	
*Gyk*	glycerol kinase	
*Hsd11b1*	hydroxysteroid 11-beta dehydrogenase 1	
*Itgb8*	integrin beta 8	
*Kcna2*	potassium voltage-gated channel, shaker-related subfamily, member 2	
*Kcnk1*	potassium channel, subfamily K, member 1	
*Luzp2*	leucine zipper protein 2	
*Mdga2*	MAM domain containing glycosylphosphatidylinositol anchor 2	
*Ncan*	neurocan	
*Negr1*	neuronal growth regulator 1	
*Nfia*	nuclear factor I/A	
*Nfib*	nuclear factor I/B	
*Nlgn1*	neuroligin 1	
*Nrxn1*	neurexin I	
*Paqr8*	progestin and adipoQ receptor family member VIII	
*Pcdh15*	protocadherin 15	
*Phka1*	phosphorylase kinase alpha 1	
*Prex2*	phosphatidylinositol-3,4,5-trisphosphate-dependent Rac exchange factor 2	
*Ptprd*	protein tyrosine phosphatase, receptor type, D	
*Ptprz1*	protein tyrosine phosphatase, receptor type Z, polypeptide 1	
*Rgs7bp*	regulator of G-protein signalling 7 binding protein	
*Slc1a2*	solute carrier family 1, member 2	
*Slc4a4*	solute carrier family 4, member 4	
*Spag5*	sperm associated antigen 5	
*Timp4*	tissue inhibitor of metalloproteinase 4	
*Tmed5*	transmembrane p24 trafficking protein 5	
*Tmem132b*	transmembrane protein 132B	
*Tmtc2*	transmembrane and tetratricopeptide repeat containing 2	
*Tshz1*	teashirt zinc finger family member 1	

**Figure 4:**
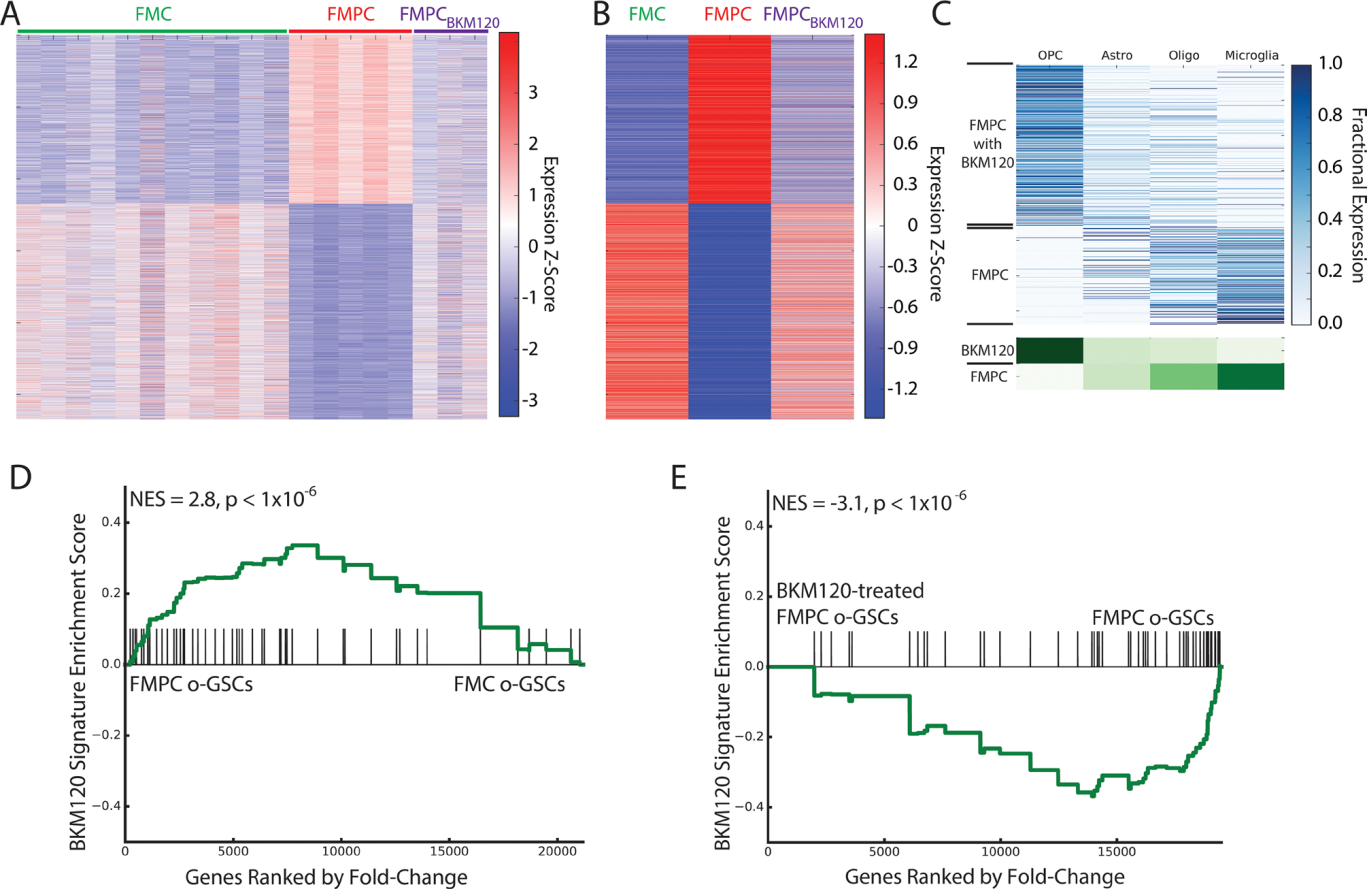
(**A**) Heatmap of RNA-Seq expression z-scores computed for genes that are differentially expressed (*p*_adj_ < 0.01, |log_2_(fold-change)| > 1) between FMPC and FMC gliomas across FMC, FMPC, and BKM120-treated FMPC gliomas. Many of the differentially expressed genes are normalized by BKM120 treatment. (**B**) Same as (A), but averaged over all samples for each group. (**C**) Deconvolution of genes that are differentially expressed between BKM120-treated and untreated FMPC gliomas. In the top panel, each row in the heatmap corresponds to a specific differentially-expressed gene and in each column corresponds to a cell type. The lower panel shows the aggregate expression in each cell type for all genes that are high in BKM120-treated and untreated FMPC tumors. The analysis shows that the treated tumors are highly enriched in OPC-like gene expression (similar to the FMC tumors) compared to the untreated tumors. (C) GSEA showing enrichment of the 50-gene BKM120 normalization signature in the RNA-Seq profile of FMPC- compared to FMC-derived o-GSCs. (**D**) GSEA showing depletion of the 50-gene BKM120 normalization signature in BKM120-treated FMPC-derived o-GSCs compared to vehicle-treated cells.

Because PTEN is a negative regulator of PI3K/AKT signaling and *Nf1* optic glioma growth [25] we hypothesized that pharmacologic inhibition of PI3K might normalize this differentially-expressed gene signature in FMPC mice. The ability of a PI3K-specific inhibitor, BKM120, to restore the FMC gene expression pattern in FMPC mice was revealed by sample-specific and average expression profiles (Figure 4A, 4B). Consistent with differential expression of OPC-associated genes in FMC tumors, deconvolution demonstrated a pronounced enrichment of OPC-associated genes in FMPC tumors following treatment with BKM120 (Figure 4C). These results demonstrate that a PI3K-regulated gene signature (Table 3) partly accounts for the differences observed between FMC and FMPC optic gliomas.

Since the heterozygous *Pten* deletion was restricted to the neoplastic cells by virtue of the fact that Cre-mediated *Pten* reduction was targeted to GFAP transgene-expressing cells containing somatic *Nf1* loss, we sought to determine whether this signature was maintained in optic glioma stem cells (o-GSCs) that derived from FMC and FMPC mouse tumors. Previously, we showed that o-GSCs from FMC mice are true cancer stem cells, with the ability to self-renew, undergo multi-lineage differentiation, (Supplementary Figure 2) and form glioma-like lesions when transplanted into naïve mice [39]. RNA-seq was performed using independent isolates of o-GSCs from at least three pools of FMC and FMPC mice (*n* = 2 mice/pool). Similar to that observed in the whole tumors, there was a relative depletion of the BKM120 normalized signature in FMC versus FMPC o-GSCs (Figure 4D), supporting a primary effect of heterozygous *Pten* deletion on the neoplastic cells in these gliomas. In addition, to determine whether BKM120 treatment normalizes this gene signature in purified o-GSCs, we treated separate pools of o-GSCs derived from FMPC mice with BKM120 *in vitro* and observed decreased PI3K/AKT activity (Supplementary Figure 3). As observed following BKM120 treatment of FMPC mice, we found the same signature to be significantly depleted in BKM120 treated o-GSCs relative to vehicle-treated controls (Figure 4E). Taken together, the BKM120-normalized gene signature behaves similarly in purified glioma stem cells and glioma tissue from the same models.

**Table 3:** Genes comprising the 50-core signature responsive to BKM-120 inhibition

Gene	Name
*Actb*	actin, beta
*Akr1a1*	aldo-keto reductase family 1 member A1
*Aldoa*	aldolase, fructose-biphosphate A
*Alyref*	Aly/REF export factor
*Arf5*	ADP ribosylation factor 5
*Arl4d*	ADP ribosylation factor-like GTPase 4D
*Arpc3*	actin-related protein 2/3 complex subunit 3
*Atp6v1f*	ATPase H+ transporting V1 subunit F
*Cct5*	chaperonin-containing TCP1 subunit 5
*Cd81*	CD81 antigen
*Cdc37*	cell division cycle 37
*Cdr1*	cerebellar degeneration related protein 1
*Cebpb*	CCAAT/enhancer binding protein, beta
*Chp2*	calcineurin-like EF-hand protein 2
*Cilp*	cartilage intermediate layer protein
*Dpysl2*	dihydropyrimidinase like 2
*Dusp1*	dual specificity phosphatase 1
*Dynll1*	dynein light chain LC8-type 1
*Eif3d*	eukaryotic translation initiation factor 3 subunit D
*Ftl1*	ferritin light polypeptide 1
*Fxyd1*	FXYD domain-containing ion transport regulator 1
*Gpr81*	hydroxycarboxylic acid receptor 1
*Herpud1*	homocysteine inducible ER protein with ubiquitin-like domain 1
*Hnrnpl*	heterogeneous nuclear ribonucleoprotein
*Ifitm2*	interferon-induced transmembrane protein 2
*Irf2bp2*	interferon regulatory factor 2 binding protein 2
*Kdelr3*	KDEL endoplasmic reticulum protein retention receptor 3
*Loxl1*	lysyl oxidase like 1
*Lsp1*	lymphocyte-specific protein 1
*Map1lc3b*	microtubule-associated protein 1 light chain 3 beta
*Mrpl12*	mitochondrial ribosomal protein L12
*Oat*	ornithine aminotransferase
*Opalin*	oligodendrocytic myelin paranodal and inner loop protein
*Pam*	peptidylglycine alpha-amidating monooxygenase
*Plat*	plasminogen activator, tissue type
*Plekhf1*	pleckstrin homology and FYVE domain containing 1
*Rasgef1b*	RasGEF domain family member 1B
*Rhoc*	ras homolog family member C
*Slc6a13*	solute carrier family 6 member 13
*Snrpb*	small ribonuclear ribonucleoprotein polypeptides B and B1
*Spon2*	spondin 2
*Sqstm1*	sequestosome 1
*Tgfbi*	transforming growth factor, beta-induced
*Tppp3*	tubulin polymerization promoting protein family member 3
*Tram1*	translocation-associated membrane protein 1
*Ubb*	ubiquitin B
*Vtn*	vitronectin
*Wdr1*	WD repeat domain 1
*Ywhaz*	tyrosine 3-monooxygenase/tryptophan 5-monooxygenase activation protein zeta
*1810011O10Rik*	unknown

**Figure 5:**
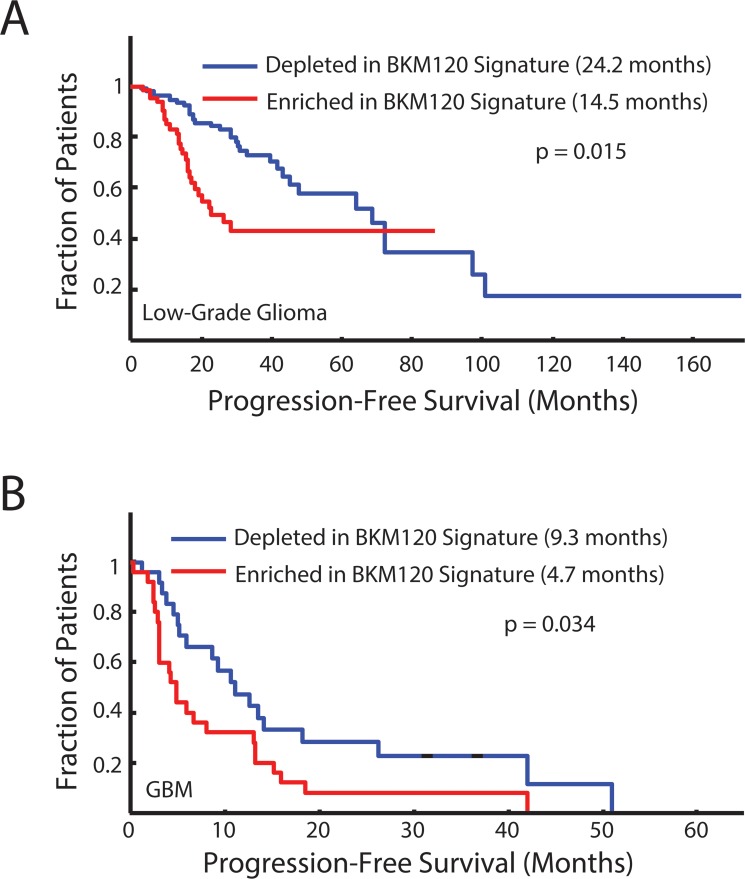
Progression-free survival curves for human low-grade gliomas (**A**) and glioblastomas (**B**) that are either highly enriched or depleted of the top 50 genes that are differentially expressed between FMPC and FMC gliomas and most significantly normalized by BKM120 treatment of FMPC gliomas. Depletion of the 50-gene BKM120 normalization signature is associated with better progression-free survival in both low-grade (*p* = 0.015) and high-grade (*p* = 0.034) human brain tumors.

One of the clinically-relevant uses of these differentially-expressed gene signatures is their application to predicting survival in patients with gliomas. We first examined the 25-gene core signature, common to all murine *Nf1* optic gliomas, and found that it did not separate patients with either low-grade or high-grade gliomas into clinically-relevant subgroups based on overall survival or progression-free survival (data not shown). However, in striking contrast, differences in patient survival were observed using an expression signature comprised of the top 50 FMPC-associated genes that were most significantly normalized by BKM120 treatment (BKM120 normalized signature). For these analyses, gene set enrichment analysis (GSEA) was employed to assess the relative expression of this gene signature across human low- and high-grade glioma surgical specimens collected and profiled by The Cancer Genome Atlas (TCGA) consortium (obtained from the Broad Institute GDAC Firehose site). In both low-grade (Figure 5A) and high-grade (Figure 5B) glioma, patients with tumors depleted in the BKM120 treatment signature exhibited significantly enhanced progression-free survival relative to those with a PTEN-enriched signature.

Taken together, these data support a model in which murine *Nf1* optic gliomas operate as an ecosystem composed of a diverse collection of cellular and acellular elements. The observation that the discovered 25-core differential gene expression pattern, which distinguishes tumor from non-neoplastic tissue, does not emanate from a single cell type (o-GSCs or microglia) argues that it represents networks of genes that are differentially expressed as a result of forming a tumor. This new ground state thus defines glioma at a molecular level, independent of gross morphological changes or alterations in cell composition or proliferation. Moreover, the established glioma gene expression profile provides a platform to evaluate potential chemotherapeutic agents, either alone or in combination, for their potential to normalize the tumor ecosystem and achieve greater treatment responses. In addition, co-existing genetic mutations targeted to the neoplastic cell compartment (*Pten* and *Nf1* inactivation) can create differential core signatures that reflect the individual contributions of these molecular events to the cancer cells, which can be used to identify prognostic signatures relevant to patient survival and the potential interpretation of successful inhibition of the regulated growth control pathways. Future studies employing these types of approaches may be instructive, not only for defining what constitutes tumor, but also for identifying a possible Achilles heel network node for therapeutic targeting.

## MATERIALS AND METHODS

### Mice

FF (*Nf1*^flox/flox^), FMC (*Nf1*^flox/mut^; GFAP-Cre) FMPC (*Nf1*^flox/mut^; *Pten*^flox/wt^; GFAP-Cre), F18C (*Nf1* gene mutation c.2041C>T; p.R681X; *Nf1*^flox/R681*^; GFAP-Cre) mice were generated as previously described [23–25]. FMOC mice were generated by intercrossing *Nf1*flox/mut mice with Olig2-Cre mice (B6-Olig2^tm2 (TVA, cre)Rth^/J; Jackson Laboratory; *Nf1*^flox/mut^; Olig2-Cre). All mice were maintained on a C57Bl/6 background and procedures performed in accordance with an approved Animal Studies Committee protocol at Washington University.

### Pharmacological studies

50 mg/kg/day minocycline (Sigma-Aldrich; PBS, vehicle) was delivered by intraperitoneal (i.p.) injection 5 days/week for two weeks [17]. 5 mg/kg/day rapamycin (Selleckchem; 5.2% Tween 80/5.2% polyethylene glycol 400 as vehicle) was administered by i.p. injection 5 days/week for two weeks [29]. 20 mg/kg/day NVP-BKM120 (Selleckchem; N-methyl-2-pyrrolidone/ polyethylene glycol 300 (10/90v/v) as vehicle) was delivered by oral gavage 5 days/week for two weeks [40]. 60 mg/kg/day carboplatin (Sigma-Aldrich; PBS as vehicle) was administered by i.p. injection every other day for 1 week. All optic nerves were harvested two weeks after the initial injection, and processed for RNAseq, immunohistochemistry and optic nerve measurements as described below.

### Optic nerve measurements

Following perfusion with Ringers solution and 4% paraformaldehyde (PFA), optic nerves were photographed and volumes measured as previously described [29].

### Immunohistochemistry

Optic nerves were microdissected and processed as described previously [41]. Antibodies used are listed in Supplementary Table 1.

### Cell culture

Optic glioma stem cells (o-GSCs) were isolated and maintained as previously described [39]. For NVP-BKM120 treatments, cells were cultured in neural stem cell medium without EGF and bFGF for 1 hour, followed by treatment with DMSO (vehicle) or 0.2 μM NVP-BKM120 for 5 hours at 37°C, and cells harvested for RNA isolation or western blotting.

### Western blotting

Cells were lysed as described previously and western blotting performed using the antibodies listed in Supplementary Table 1 [42, 43].

### Immunocytochemistry

o-GSCs were adhered to fibronectin (50 μg/ml)/poly-D-lysine (10 μg/ml)-coated surfaces and cultured in neural stem cell medium supplemented with N2, B27, EGF and bFGF for 24 hours at 37°C and fixed with 4% PFA for immunostaining. o-GSCs were also differentiated as previously described [39], fixed with 4% PFA, and immunostained with the antibodies listed in Supplementary Table 1.

### RNA extraction and mRNA isolation

RNA was extracted from Trizol-preserved tissue and cell lysates using isopropanol precipitation. Each total RNA sample was re-suspended in 20 uL of nuclease-free water supplemented with 0.5 U/uL RNase inhibitor (SUPERaseIN, Life Technologies). A 5′-biotinylated, LNA-enhanced oligo(dT) capture probe (Exiqon) was used to isolate poly(A)+ mRNA as described previously [44].

### Library construction and sequencing

RNA-Seq libraries were constructed using SMARTer Stranded RNA-Seq kit (Clontech Laboratories) according to the manufacturer's instructions. mRNA was fragmented for 4 minutes at 94°C and cDNA libraries were purified twice using AMPure XP beads (Beckman) prior to PCR enrichment. After PCR enrichment, the resulting sequencing libraries were quantified using a Bioanalyzer (Agilent) and Qubit (ThermoFisher). The libraries were pooled and sequenced on an Illumina NextSeq 500 using 75-cycle High Output Kits.

### RNA-Seq data processing

Prior to sequence alignment, the first five nucleotides from the 5′-end of each read were clipped to eliminate the GC-rich, template-switching sequence inserted during reverse transcription. The reads were aligned to the mouse genome and transcriptome (mm10, USCS annotation from Illumina iGenomes) using Tophat 2 [45]. HTSeq was used to quantify the number of reads associated with each gene and DESeq2 for differential expression analysis [46]. The RNA-seq data will be deposited in GEO.

### Deconvolution of cell type-specific gene expression and gene set enrichment analysis

Computational deconvolution of cell type-specific gene expression was accomplished using the methods described in our previous studies [27, 28] with one improvement. As cell type-specific markers, we used *Cspg4* (OPC), *Gfap* (astrocyte), *Mog* (oligodendrocyte), *Rbfox3* (neuron), and *Aif1* (microglia). Gene set enrichment analysis was conducted using the GSEA software package available from the Broad Institute. For all of the GSEA presented here, we used pre-ranked/classical mode to compute enrichment scores.

## SUPPLEMENTARY FIGURES AND TABLE


