# Supplementary material for: Whole tumor RNA-sequencing and deconvolution reveal a clinically-prognostic PTEN/PI3K-regulated glioma transcriptional signature
Source: Oncotarget. 2017 Apr 18;8(32):52474–87. doi: 10.18632/oncotarget.17193 (PMC5581044; doi:10.18632/oncotarget.17193)
Supplement: Supplementary file 1 [file oncotarget-08-52474-s001.pdf]

Whole tumor RNA-sequencing and deconvolution reveal a clinically-prognostic PTEN/PI3K-regulated glioma transcriptional signature

Supplementary Materials

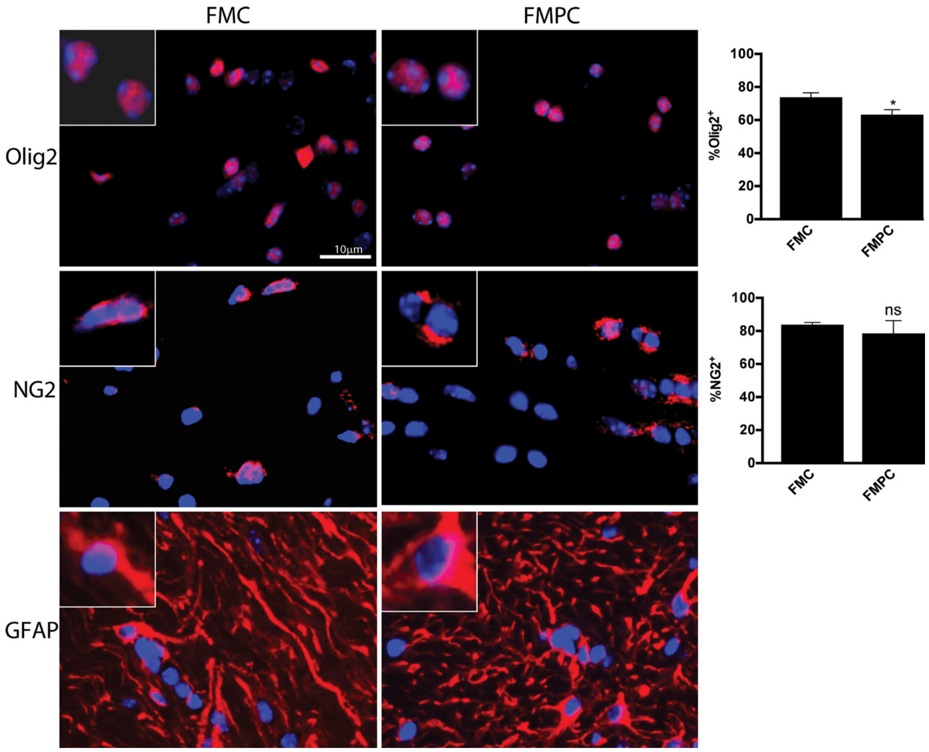

Supplementary Figure 1: Immunostaining of FMC and FMPC optic gliomas reveals similar expression patterns and numbers of Olig2⁺, NG2⁺ and GFAP⁺ cells. There was a slight reduction in the percentage of Olig2⁺ cells in the FMPC optic gliomas. *$p = 0.0304$. ns, not significant ($p = 0.5441$). Scale bar, 10 µm.

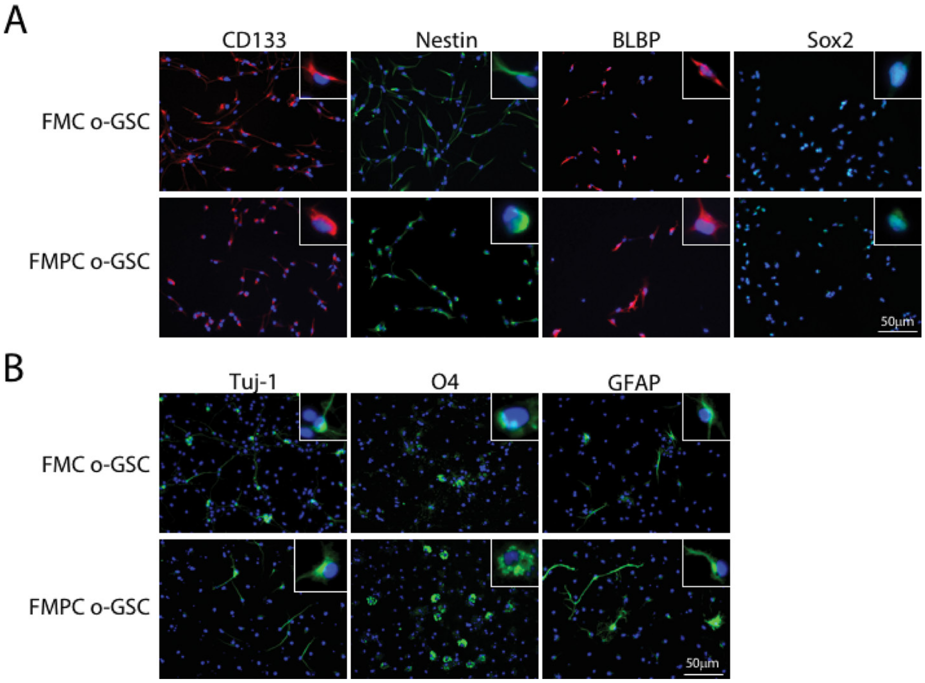

Supplementary Figure 2: Characterization of o-GSC cultures. (**A**) FMC and FMPC optic glioma stem cell (o-GSC) cultures similarly express stem cell markers, including CD133, nestin, BLBP, and Sox2. (**B**) FMC and FMPC o-GSCs are both capable of multi-lineage differentiation into Tuj-1$^+$ neurons, O4$^+$ oligodendrocytes, and GFAP$^+$ astrocytes. Scale bar, 50 µm.

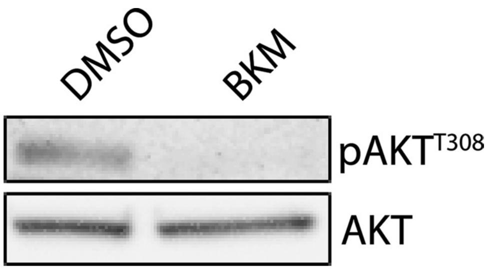

Supplementary Figure 3: Western blotting shows that treating FMPC o-GSCs with BKM120 inhibited PI3K/AKT activity. DMSO serves as the vehicle control. BKM, BKM120.

Supplementary Table 1: Antibodies used

Antibody	Host	Source	Dilution
AKT (WB)	Rabbit	Cell Signaling, 9272	1:1000
anti-mouse HRP (IHC)	-	Vector, BA-9200	1:200
anti-rabbit HRP (WB)	-	R&D, 7074	1:5000
BLBP (ICC)	Rabbit	Millipore, ABN14	1:200
CD133 (ICC)	Rat	Millipore, MAB4310	1:100
GFAP (ICC)	Mouse	Millipore, MAB3402	1:500
Ki67 (IHC)	Mouse	BD Pharmingen, 550609	1:500
Nestin (ICC)	Mouse	Millipore, MAB353	1:200
O4 (ICC)	Mouse	Millipore, MAB345	1:100
phospho-AKT^{T308} (WB)	Rabbit	Cell Signaling, 9275	1:200
Sox2 (ICC)	Rabbit	Millipore, AB5603	1:100
Tuj-1 (ICC)	Mouse	Biolegend, MMS-435P	1:1000

WB: Western Blot; IHC: Immunohistochemistry; ICC, immunocytochemistry; HRP: horseradish peroxidase
Secondary antibodies conjugated to Alexa Fluor 488/568 (ICC) were purchased from Thermo Fisher Scientific.
